# Memory in Leopard Geckos (*Eublepharis macularius*) in a Morris Water Maze Task

**DOI:** 10.3390/ani15142014

**Published:** 2025-07-08

**Authors:** Eva Landová, Aleksandra Chomik, Barbora Vobrubová, Tereza Hruška Hášová, Monika Voňavková, Daniel Frynta, Petra Frýdlová

**Affiliations:** Department of Zoology, Faculty of Science, Charles University, Viničná 7, 128 00 Prague, Czech Republic; eva.landova@natur.cuni.cz (E.L.); chomika@natur.cuni.cz (A.C.); barbora.zampachova@seznam.cz (B.V.); hasigora@seznam.cz (T.H.H.); monika.vonavkova@seznam.cz (M.V.); daniel.frynta@natur.cuni.cz (D.F.)

**Keywords:** cognition, memory, Morris water maze, orientation, reptile learning, spatial navigation, Squamata

## Abstract

The spatial orientation of mammals and birds has been intensively studied for many years, but the cognitive mechanism of spatial orientation and memory used by squamates remains poorly understood. Our study evaluated the learning and memory abilities of leopard geckos (*Eublepharis macularius*) in an adapted Morris water maze. The animals learned during the training phase consisted of 20 trials. To assess long-term memory, we retested geckos twice after several months. The geckos remembered the learned information in a short re-test after two months, but after four months, they required retraining to find the platform. We hypothesise that the duration of memory corresponds with short-term changes in semi-desert environments within one season, while disruption of memory performance after a six-month gap may simulate the more extensive seasonal change in spatial relationships in their natural environment. Moreover, during the winter period, geckos exhibit low activity, which can be connected with decreased frequency of foraging trips. Therefore, the memory loss after four months may reflect the low level of memory jogging. The motivation during the experiment was the crucial parameter of learning and memory processes. In later phases, geckos were less motivated to perform the task. Finally, they relearned the spatial orientation task, but they moved more slowly as the experiment progressed.

## Simple Summary

Understanding how reptiles like lizards remember and navigate their surroundings helps us learn more about animal intelligence. We tested how well leopard geckos can learn and remember the path to find a hidden platform in a water maze task. They remembered the task after two months, but after six months, their memory had faded, and they needed to relearn it. Some geckos showed different navigation strategies and levels of motivation, which affected their performance. These results show that leopard geckos are capable of learning and remembering, but their memory may be adapted to the seasonal changes in their natural environment. Our findings provide new insight into reptile cognition and highlight the importance of individual differences in learning and memory.

## 1. Introduction

Spatial memory, which includes both short-term and long-term memory, is crucial for animals’ survival and adaptability. Short-term memory allows animals to make immediate decisions about their environment, such as recognising the position of potential threats or locating food sources in real time [1,2]. Long-term memory, on the other hand, enables them to recall important landmarks or remember migratory routes, territory boundaries, and social interactions over extended periods [3,4]. The ability to retain and recall this information is vital not just for spatial orientation, but for broader ecological interactions, including foraging, migration, and social bonding [5]. For example, elephants rely on both long-term spatial and social memory to navigate vast landscapes, recall water sources, and maintain social cohesion within their herds [6,7]. Similarly, pigeons use a combination of spatial memory and cognitive mapping to navigate back to their home lofts, even from hundreds of kilometres away, by relying on various environmental cues like the sun, magnetic fields, and olfactory signals [8,9]. In birds like corvids, food caching is not only a function of spatial memory, but also requires the ability to recall where and when they hid their food, illustrating how ecological pressures shape both short and long-term memory capabilities [10,11]. Short-term and long-term memory in reptiles differ structurally and functionally. Short-term memory involves the medial cortex [12] and dorsal ventricular ridge [13] and is associated with transient synaptic changes [14]. However, long-term memory is associated with long-term potentiation, a process that strengthens synapses over time and involves synaptogenesis [15]. Long-term memory storage occurs in brain regions like the medial and lateral cortex [16]. Good memory performance is particularly important for long-lived species such as turtles [17], monitor lizards [18], and even other lizards [19]. Spatial orientation in reptiles, like memory, is largely dependent on the medial cortex [20] and dorsomedial and dorsal cortex [21].

Reptiles, particularly members of the order Squamata, which includes lizards, snakes, and worm lizards, have been studied far less in terms of spatial memory. Squamates are a diverse group of reptiles found in a variety of habitats, from deserts to rainforests. Unlike mammals and birds, where spatial memory research is extensive, studies on squamates remain limited and show varied results. There are several reviews covering advances in understanding reptile cognitive abilities, including spatial cognition (for reviews, see [22,23,24]). The studies concentrated on testing the spatial learning and memory abilities of squamates using natural behaviours of many species, such as hiding in shelters or predator avoidance [25]. Flexible spatial learning was demonstrated under semi-natural conditions in the golden water skink (*Eulamprus quoyii* [26]). The experimental design involved simulated predatory attacks to encourage the lizards to learn and adapt to their environment. Animals went under a spatial learning task and a reversal learning task, showing that 32% of lizards successfully learned both the initial and reversal tasks within 10 days. These results demonstrate that *Eulamprus quoyii* can flexibly adapt their spatial learning in response to changing environmental cues. Some of the new procedures use methodology developed for rodents, modified to reptile needs. Examples of such studies may be an adaptation of the Barnes maze for reptiles tested in common side-blotched lizards (*Uta stansburiana* [27]), corn snakes (*Elaphe guttata guttata* [28]), and spotted pythons (*Antaresia maculosa* [29]). Since lizards rely on external heat sources for activity, the maze was placed on a temperature-controlled surface to ensure optimal performance. Unlike rodents, which seek enclosed burrows, the reptiles were provided with a natural shelter. While rodents often rely on scent cues, reptiles depend more on visual landmarks. Some of the reptile species are able to navigate in adapted mazes like the Morris water maze (MWM), which has been successfully used for studying spatial orientation learning in the Italian wall lizard (*Podarcis sicula* [30]) and leopard gecko (*Eublepharis macularius* [31]).

Reptiles employ a variety of strategies for small-scale navigation, ranging from direct goal identification using specific features to the use of spatial memory to understand object relationships. Studies such as that by Landová and colleagues [31] on leopard geckos in the MWM demonstrate their adeptness at using cues placed within the arena, as well as those scattered throughout the experimental room. Researchers systematically varied the number of reliable cues within the arena (proximal cues) and included a distinct cue within the room (distal cue) to observe how these changes affected the geckos’ navigation strategies. The geckos demonstrated learning capabilities, indicating an ability to adapt to changes in their environment. No single manipulation of proximal or distal cues resulted in a complete loss of orientation across all subjects, suggesting a robust spatial learning ability. The study also noted significant individual variability in motivation and the quality of spatial information learned, highlighting differences in cognitive strategies among the geckos. Another kind of navigation was tested by Noble and others [26], where they examined golden water skinks that displayed flexible learning by adjusting their navigation when the environment changed, supporting allocentric spatial memory. Moreover, Holtzman and others [28] proved that corn snakes learned to navigate using visual and tactile landmarks, displaying allocentric learning through external spatial relationships. Additionally, research by Kundey [32] suggests that reptiles may even incorporate the shape of the arena itself into their navigation strategy. The geckos demonstrated the capacity to recognise and respond to changes in both the shape of the arena and the placement of objects within it, indicating that they utilise environmental geometry as part of their navigation strategy. Regardless of the method reptiles use to gather spatial information, a fundamental question remains: how long do they remember the learned information, and can they effectively use it over prolonged periods?

Turtles are more commonly used in studies of learning and memory than other ectotherms. It is well known that despite various cognitive abilities, including social cognition [33,34,35], they have limited short-term memory [36], although they make up for that with their great long-term memory [37,38]. The turtle’s memory abilities are tightly related to their survival and longevity ([39], but see also for the rate of ageing [40]). Studies on red-bellied cooters (*Pseudemys nelsoni*) have shown that turtles can retain learned tasks for up to 36 months, while red-footed tortoises (*Chelonoidis carbonarius*) have been able to recall associations between visual cues and food rewards after 18 months [41]. Similarly, Aldabra (*Aldabrachelys gigantea*) and Galápagos tortoises (*Chelonoidis* cf. *nigra*) exhibited memory retention for up to nine years, though some relearning was required [42]. These findings highlight the importance of long-term memory in species with extended lifespans, where learning plays a critical role in survival and resource acquisition.

Studies on crocodilians also provide insights into memory in long-lived reptilian species. Research on American alligators (*Alligator mississippiensis*) showed their ability to navigate toward water sources in a controlled arena, demonstrating spatial learning based on environmental cues [43]. Further studies on American crocodiles (*Crocodylus acutus*) indicated that both alligators and crocodiles can perform discrimination tasks that involve spatial memory and cue learning [44].

Studies on memory and learning abilities in Squamata have provided valuable insights into the cognitive capabilities of this reptilian order. While some species exhibit clear evidence of spatial learning and memory, others show limited or no spatial memory, especially in relation to spatial navigation tasks. In several studies, spatial memory in squamates appears to be absent or weak. For example, Bosc’s fringe-toed lizard (*Acanthodactylus boskianus*, [45]) and the little striped whiptail (*Cnemidophorus inornatus*, [46,47]) failed to demonstrate significant spatial memory in maze cues. However, other studies in squamates have highlighted species that do possess spatial memory. For example, corn snakes (*Elaphe guttata guttata*) were tested using the Barnes Maze, a common task for assessing spatial memory in reptiles. In these studies, Holtzman [28,48] demonstrated that corn snakes were able to learn the task after four days of testing. They showed clear spatial learning ability, using external cues to navigate the maze, suggesting that spatial memory in this species may be used for navigation and finding food or shelter in their natural environment. Similarly, LaDage and others [27] provided evidence of spatial memory and learning abilities in side-blotched lizards (*Uta stansburiana*), which were tested in a modified Barnes Maze. These lizards were able to learn to navigate the maze, although their strategies varied significantly between individuals. This variation suggests that while spatial memory is present in side-blotched lizards, there may be individual differences in the strategies used for spatial orientation, possibly reflecting differences in learning styles or ecological niches. Another notable study on squamates’ learning abilities focuses on monitor lizards (family Varanidae), known for their problem-solving and cognitive flexibility. In one study, Loop [49] examined Bengal monitors (*Varanus bengalensis*) in a problem-solving task where the lizards had to press a button to access food. All four of the tested Bengal monitors successfully learned to perform the task. Other studies [50,51] extended this work to other monitor species such as *Varanus rudicollis*, *V. prasinus*, and *V. mertensi*, which were able to learn how to open a container to retrieve food. These tasks were more related to discrimination learning (learning to distinguish between objects or actions to obtain rewards) than to spatial navigation.

Overall, there is a clear gap in research on spatial memory and spatial cognition in squamates, especially compared to mammals and birds. While some species, such as corn snakes and side-blotched lizards, have shown strong evidence of spatial learning and memory, other species exhibit only discrimination learning or demonstrate little spatial memory. This variation underscores the need for further studies, particularly in how squamates might use environmental cues and cognitive flexibility to navigate their environments.

The leopard gecko (*Eublepharis macularius*; Blyth, 1854) is one of the most widely studied species within the Squamata lineage [52,53]. It is successfully kept and bred under laboratory conditions and has been used in research on body growth [54], hybridisation [55], physiology and brain function [56,57], as well as locomotor performance during periodic movement [58]. It can be found in India, Pakistan, Afghanistan, and Iran in desert and semi-desert areas [59]. Leopard geckos possess movable eyelids and are notable for their exceptional longevity [60]. In captivity, they can live more than 28 years, as documented in the AnAge Database [61]. Chemical communication is crucial for nocturnal geckos, with olfaction playing a more significant role than vision in recognising conspecifics and potential predators, such as snakes [62]. This dominance of the chemical modality is further supported by research on another nocturnal gecko (*Gekko gecko*), where the ability to remember odours helps in both environmental navigation and social interactions [63]. While chemical cues are the primary sensory modality, leopard geckos also effectively use visual information. They employ a variety of navigation strategies, ranging from orientation by a single mark (cue learning) to allothetic orientation utilising relationships between multiple marks [31] or navigation by arena shape [32]. However, it is still not known if they have long-term memory and how efficiently the animals can use learned information after a longer time, e.g., several months. As long-lived animals, they may be capable of retaining memories for many months or even years. The reliability of the cues, as well as the individual’s preference for and experience with a particular set of cues (or navigation strategy), may be important in ensuring the persistence of long-term memory. While moving through the environment, even reptiles may use complex navigation strategies that combine information from distal cues (prominent landmarks in the environment that indicate the individual’s global position, in laboratory experiments, these are often marks on the walls of a room), local cues (located in the vicinity of the animal, often close to the target, in arena experiments, these are often found on the arena wall), or navigational compasses utilizing more global cues (sun compass, stellar compass, magnetic compass, etc. [24]). A notable example is the painted turtle (*Chrysemys picta*), which by the age of four, learns to migrate from temporary to permanent water sources based on visual and chemical markers during a critical period (reviewed in [64]). Adult turtles depend on long-term memory to navigate this migration, preferring to use local markers [64,65]. In the absence of local markers or experience (i.e., juveniles or inexperienced adults), all individuals may use global cues, such as simulated reflected light from the water surface. However, experienced turtles prefer learned local cues to novel global markers. Therefore, even when they were capable of flexible and complex navigation, they relied on the reliable, experience-based cues they had already learned [66].

In our previous study [31], we were able to successfully use the MWM task to study the visual–spatial orientation in the adult leopard gecko (*E. macularius*). In this task, animals are not able to use the chemoreception that is usually important for them. We tested the spatial performance of the geckos in detail, and we found that they used both distant as well as close arena cues for orientation. The cues that the leopard geckos exploited were highly individual, depending on the reliability of the cues and preferred individual strategy. In the current study, we ask how long spatial information persists in a gecko’s long-term memory. Specifically, we wanted to test their long-term memory of spatial information in two periods after the end of the training: first, after two months from the end of the training phase, which simulates short-term changes within one season (breeding season), and second, after six months from the end of the training phase (four months after retraining), which simulates long-term changes in their natural environment. After six months, environmental changes such as loss of visual landmarks or temperature fluctuations might result in memory loss. Geckos might need to rely on other strategies, such as exploring new environmental cues or adapting to altered cues.

## 2. Materials and Methods

### 2.1. Experimental Animals

We studied adult leopard geckos (*Eublepharis macularius*, Eublepharidae) aged between 2 and 5 years, which were predominantly females with only one male. The experimental animals were individuals from the laboratory population, as well as descendants (first and second generation) of wild animals caught in the natural environment (Pakistan), for further details see [54,55]. Leopard geckos have temperature-dependent sex determination [67], and the incubation temperature may also affect their behavioural traits [68,69]. To avoid the effects of incubation and attitudes towards the sex of experimental animals in favour of non-territorial females, we set the incubation temperature to 28.5 °C ± 0.5, which is close to the temperature preferred by the females of *E. macularius* for egg laying (approximately 29 °C; [70,71]). At first, we used 56 naïve animals in the training phase. Nevertheless, some animals did not swim actively and rather adopted a passive floating strategy to save energy. To eliminate this behaviour, we gently poked the animal with a wooden stick at the base of the tail to simulate a predator attack [72,73]. The number of these touches during the trial was recorded. It is negatively correlated with the animals’ willingness to search for the platform. In total, 42 individuals (41 females and 1 male) finished the initial training and passed the first memory test. Finally, we randomly selected 28 animals (27 females and 1 male) for the second memory test. Before testing, geckos were weighed and scanned. From scans, we measured the length of animals (SVL—Snout-Vent Length) and tail thickness at the widest point. Leopard geckos store fat and fluids in their tails; the thickness of the tail is directly related to their condition (for assessing body condition in leopard geckos, see [74]). From these data, we determined whether the condition of the tested animals was negatively affected by the tests and whether the weight of the animals and the tail thickness were reduced during the experiment. All experimental animals were weighed and measured regularly to assess their actual body size and condition (for data, see Supplementary File S1).

### 2.2. Housing of Animals

Animals used in experiments were kept in the same conditions. They stayed in one room with natural light mode. The room temperature ranged from 26 to 30 °C, using the heating cables and the central heating in the winter months. The animals were housed individually in glass terrariums (30 × 30 × 20 cm). Animals had access to food (crickets and larvae of the beetle *Tenebrio molitor*) and water. The insects were dusted with vitamins and minerals (UniVIT, Olomouc, Czech Republic); AD3 and E vitamins (Nutri Mix, Trouw Nutrition Biofaktory, Prague, Czech Republic) were provided weekly.

### 2.3. Apparatus

**Figure 1:**
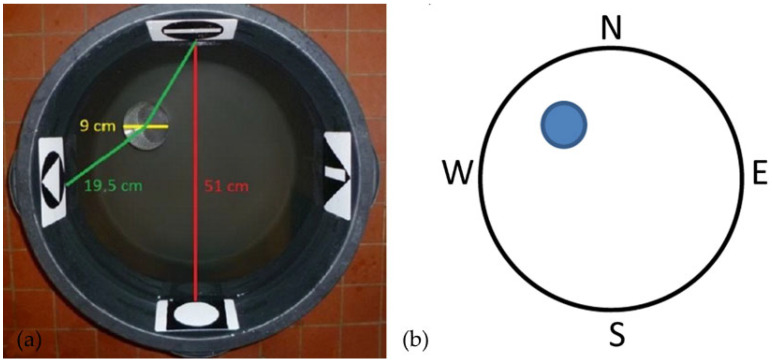
The experimental arena for MWM testing was adapted for small reptiles. A transparent plastic cylinder was under the water as a platform. Four artificial markers (arena cues) were placed at the edge of the arena (**a**). Diagram of the experimental arena with orientation according to the four cardinal points (**b**). The blue circle represents the platform. Abbreviations: N—North, S—South, E—East, and W—West.

All parts of the experiments were carried out in the Morris water maze (MWM [75]) modified to the needs of small reptiles (Figure 1a and Figure S1). The same test was used in another study [30] to explore compass and other orientation mechanisms in the ruin lizard (*Podarcis sicula*). Employing a modified MWM is highly advantageous when testing reptiles, as it prevents the presence of chemical cues and thermal gradients that could confound behavioural responses. We used a black plastic arena (51 cm diameter) with a transparent plastic cylinder as a platform (9 cm diameter). The size of the arena used was half the size of what is used for mice [76]. Water in the arena was heated to 28 ± 1 °C. The height of the water level was set so that the platform was 2 cm under the surface (the total height of the water column was 21.5 cm). For the needs of spatial orientation, four artificial markers were placed at the edge of the arena. These arena cues consisted of combinations of simple monochrome geometric shapes and represented the cardinal directions due to the orientation of the arena towards the viewer: N—North, S—South, E—East, and W—West (Figure 1b). Moreover, animals can also use more distant types of cues in the room (walls, doors, etc.). All phases of the experiment were recorded on a video camera (Sony DCR-SX15, Sony Central and Southeast Europe, Prague, Czech Republic), which was above the center of the arena (140 cm) on the rack attached to the ceiling.

### 2.4. Initial Training

During the first phase of the experiment, we tested whether the animals were able to swim actively and find the platform. The time and path needed should decrease during the process of learning. The training consisted of 20 trials (maximum one 8 min trial per week). For further statistical evaluation, we called the first three training trials S-training (Start, trials 1–3) and the last three training trials F-training (Finish, trials 18–20). The criterion of successful learning was a significant statistical difference in latency and/or path length between S-training and F-training. Geckos were released into the arena in semi-random starting positions from four directions (5 × N, 5 × S, 5 × E, 5 × W). This starting position is not equidistant from the platform (see Figure 1b). We accounted for this so-called short (N and W) vs. long (S and E) starting position parameter in the analysis. We excluded occasional trials with more than 30 touches per individual during one experiment and repeated those trials the next time.

### 2.5. Long-Term Memory Test for Spatial Orientation

**Figure 2:**
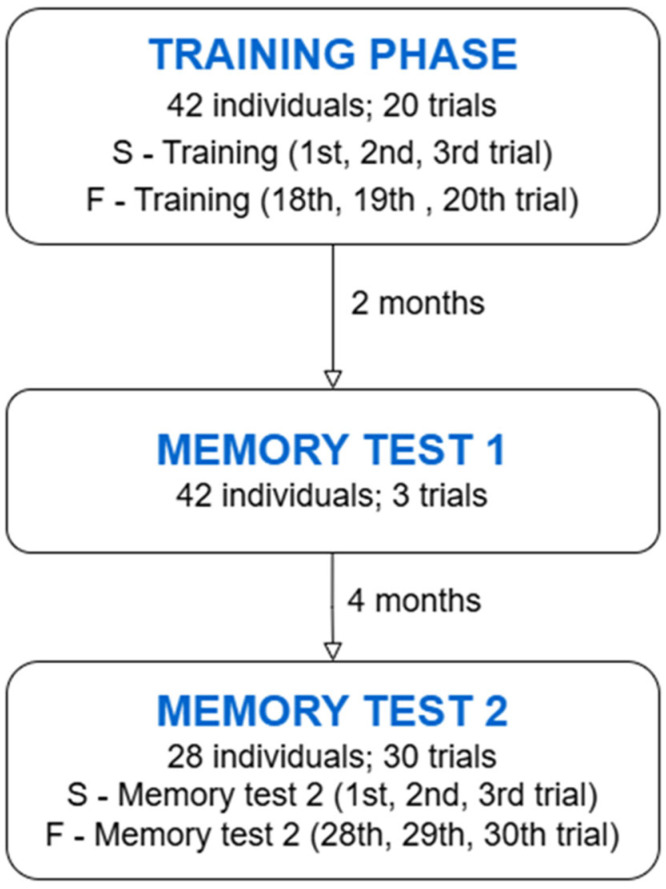
The diagram of experimental design with details about the number of animals, trial names, and numbers of experimental animals.

We tested long-term memory twice to examine the memory of leopard geckos (for the schedule of the experiment, see Figure 2). The procedure was identical to the training phase. The first memory test (Memory test 1) was two months after the termination of initial training. It included 3 trials in a max of 8 min. The second memory test was after the next four months (six months after the termination of initial training). We started with retraining (28 individuals) to test if animals can remember how to find the platform and if they are learning. The total number of trials was 30 in a maximum of 8 min. For further statistical evaluation of memory, we called the first three retraining trials S-Memory test 2 (Start, 1–3 trials) and the last three retraining trials F-Memory test 2 (Finish, 28–30 trials).

### 2.6. Behavioural Analysis

In all trials, we measured the latency (in seconds) that the gecko needed to successfully reach the platform (and whether it was found) manually by stopwatch. We scored the number of touches needed if the gecko used the floating strategy. Program Ethovision XT 11.5 [77] was used to calculate the length of the travelled path (in cm). The high dynamic brightness model was the best to use (for this purpose, animals were always marked with a non-toxic white colour on the head). This program reads the track and time spent in the arena directly from the video. Unfortunately, the program sometimes did not stop measuring the time when the gecko reached the platform, which resulted in a poor correlation between Ethovision and manually measured time. This was the reason why we used directly measured stopwatch time in all analyses. Moreover, the latency to reach the platform may be higher in animals that adopted the floating strategy or shorter in fast-moving animals. Thus, the correlation of path length with latency is not so tight (r = 0.723). We assume that the path length is more suitable for the evaluation of the learning process in our experiment. Additionally, we calculated the velocity (cm/s) as the path length divided by the latency and used it as another dependent variable in our analysis.

### 2.7. Statistical Analysis

The values of path length, latency, and velocity were log-transformed using the natural logarithm (ln) to ensure normality of the residuals. The data were analysed using linear mixed-effects models (LMMs) as implemented in the functions lme in the package nlme [78], and estimated marginal means were used for post hoc comparisons between groups in the package emmeans [79] in R 4.0.5 [80]. For the evaluation of learning and memory processes, we used path length, latency and velocity as the dependent variables, the starting position (short/long), number of touches, body weight, and the training phase (S-training, F-training, Memory test 1, S-Memory test 2 and F-Memory test 2) as fixed effects and animal ID as a random factor. The reference level for the comparison of training phases in the LMMs was the S-training phase. The reference level for the memory tests was the F-training phase. Additionally, we performed pairwise comparisons of all the phases using the Tukey post hoc test. All data are included in this published article in its Supplementary File S1.

## 3. Results

**Figure 3:**
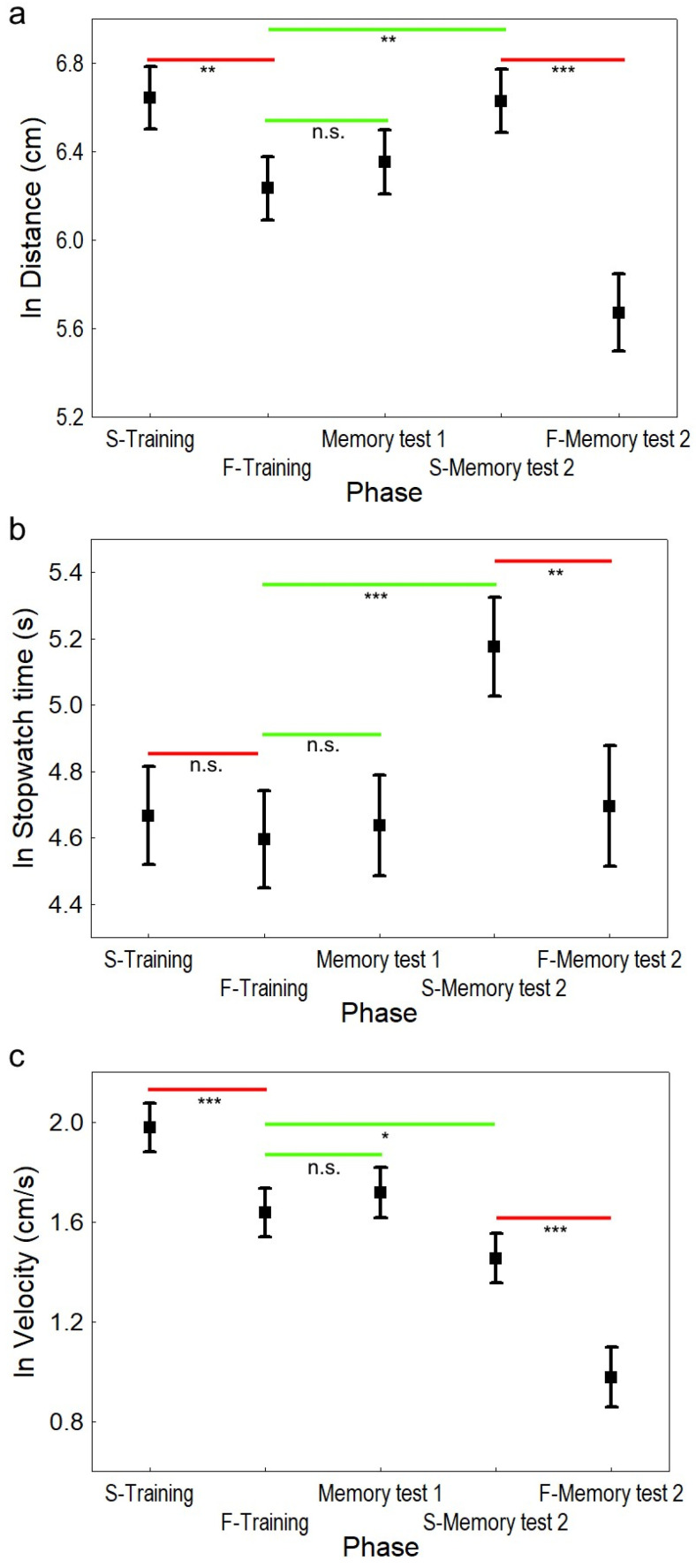
The comparison of mean path length, latency, and velocity across training phases and memory tests (mean ±95% confidence intervals of the mean). Geckos were able to learn the spatial task in the Morris water maze. (**a**) The log-transformed mean path length (ln distance) was significantly shorter in the final part of the training (F-training) in comparison to the initial training (S-training). Geckos were also able to keep the learned information after two months (Memory test 1), demonstrating long-term memory capabilities. (**b**) The log-transformed latency to find the platform (ln Stopwatch time) does not fully correspond to the path length during the trials. It is longer in the last series of the entire experiment, indicating that it reflects animals’ motivation rather than learning and memory processes. (**c**) The velocity of geckos while searching the platform can reflect the tendency to use alternative motoric strategies. The important significances of learning are depicted by red colour, while short-term and long-term memory capabilities are depicted by green colour and asterisks (* *p* < 0.05; ** *p* < 0.001; *** *p* < 0.0001, n.s. *p* > 0.05).

We found that geckos were able to learn spatial tasks. The path length was shorter (Figure 3a) in nearly all phases in comparison to the start of training (S-training). For the model with path length as the dependent variable, the starting position (F = 3.593, df = 536, *p* = 0.0586) and body weight (F = 1.160, df = 536, *p* = 0.282) were not significant and were not included in the final model. The significant variables were the number of touches (F = 280.490, df = 538, *p* < 0.0001) and the phase of the training (F = 24.157, df = 538, *p* < 0.0001). The number of touches was positively associated with the path length. We compared the different phases to the reference value (S-training). All phases had significantly shorter path lengths than in S-training, except the start of the retraining (S-Memory test 2) after six months from the initial training (*p* < 0.1; for model contrasts, see Table 1, and for model coefficients, see Table 2).

Surprisingly, the latency of the initial phase of training (S-training) was not signif-icantly longer than in the following phases of the experiment (Figure 3b). For the model with latency (stopwatch time) as the dependent variable, the statistically significant varia-bles were starting position (F = 5.406, df = 536, *p* = 0.0204), number of touches (F = 350.221, df = 536, *p* < 0.0001), phase of training (F = 10.228, df = 536, *p* < 0.0001), and body weight (F = 6.729, df = 536, *p* = 0.0097). The number of touches had a positive effect on the latency; the time was shorter for the short type of starting position. The latency to find the platform in phase S-Memory test 2 was significantly longer than in the S-training phase (*p* < 0.01), while all other phases were not significantly different from the reference value (for model contrasts see Table 3, for coefficients see Table S1 in Supplementary File S2).

Analysis with velocity as the dependent variable gives similar results (see Figure 3c and Tables S2 and S3 in Supplementary File S2) as analysis with latency and path length.

## 4. Discussion

Studies that experimentally tested spatial learning and memory in reptiles provided mixed results. While some found that certain species make use of complex visual cues for spatial learning [24], others reported no such conclusions [81]. Our results demonstrate that leopard geckos are capable of spatial learning and retaining the acquired information for at least two months. However, after a longer delay of four months, their performance declined, suggesting limitations in long-term memory retention without reinforcement.

In this study, we proved that another species of squamates, the leopard gecko, can learn to solve a spatial task in the adapted MWM over 20 trials. The animals improved their performance over the training phase, taking a shorter path to locate the platform at the end of training and in the final part of the second memory test (F-Memory test 2, six months after the initial training) compared to the first trials. Time to find the platform did not significantly differ at the end of the training phase and during the first memory test (after two months from initial training). Geckos were still able to reliably locate the plat-form after a two-month hiatus. However, after the next four months, they had forgotten the learned spatial information, and they had to relearn the position of the platform. Geckos needed a longer time to complete the task during the first part of the second memory test (S-Memory test 2) compared to the other phases, but they were able to remember and reach the platform in a significantly shorter time in the last part of the testing. We hypothesise that the path length is a more accurate parameter as it better reflects the learning abilities of geckos in MWM spatial tasks. Latencies are more influenced by the motivation of the tested animals. The velocity of animals throughout the experiment has a downward trend; it significantly decreased in the last trials of the second memory test (F-Memory test 2) when compared to the other testing parts. We do not interpret this as a memory loss but rather as a decrease in motivation during the course of the experiment. During the experiment, we observed that some geckos adopted a floating strategy, i.e., remaining motionless on the water’s surface. This behaviour could indicate a loss of motivation or, alternatively, serve as an energy-saving strategy. Despite their crucial role in cognitive task performance, only a few studies have focused on motivation levels in such a context [82]. To prevent passivity during the experiment, we gently stimulated the geckos with a stick to encourage movement and active searching for the platform. The analysis revealed that the number of touches was always a significant factor influencing geckos’ performance in the experiment.

This study indicates that leopard geckos have long-term memory, but the recall of spatial information decays over time. Their performance declined after six months, requiring retraining. If we relate the information obtained in our experiment to changes in their natural environment, it is clear that a two-month memory would cover their breeding season [83]. Loss of the spatial information after a longer period is more consistent with inter-seasonal changes and overcoming the cold season. Theoretically, geckos would be able to relearn spatial relationships each season after their semi-desert environment has been transformed by seasonal rains during the winter and spring seasons [84]. However, our hypothesis of ecological relevance would need to be tested under natural conditions.

Long-term procedural memory (memory of how to perform specific skills) of a problem-solving task was demonstrated in a small sample of squamate lizards, e.g., several individuals of monitor lizards (*Varanus prasinus* and *V. mertensi*) and the beaded lizard *Heloderma charlesbogerti* [85]. After a 20-month hiatus, they showed shorter latencies and required fewer trials to fulfil the criterion in a specific problem-solving task compared with the initial stage of training [85]. Monitor and beaded lizards are larger reptile species. Although their brains have significantly lower neuronal densities compared to birds and mammals, larger-bodied species still possess a higher absolute number of neurons than smaller ones, which may contribute to a greater capacity for long-term memory [86]. Leopard geckos have worse long-term memory compared to both monitor and beaded lizards. While they are also long-lived, they are a relatively small species compared to the aforementioned, and thus have a smaller absolute number of neurons in the terminal brain [86], which could theoretically be linked to their poorer capacity for long-term memory. They are also inferior to turtles in terms of long-term spatial memory, although this comparison is limited by the fact that turtles are not squamates and differ significantly in longevity. While geckos live up to 28 years in captivity, turtles live much longer (e.g., 80 years in *Caretta caretta*, [87]). One example of long-term memory for spatial information can be seen in sea turtles, which return to the place where they were born. Adult loggerhead sea turtles (*Caretta caretta*) return to the area near their natal beach after maturation and thus probably remember this location throughout their entire life [88]. Initially, hatchlings orient themselves according to light sources such as the moon, stars, or artificial lights, using idiothetic navigation to swim against the direction of the waves after emerging from the nest. Once in the ocean, the young turtles transfer their initial seaward heading to their magnetic compasses, enabling them to maintain an offshore course even when they can no longer see land (reviewed in [89]). The spatial orientation of hatchling turtles is determined by innate behavioural patterns, and an initial exposure to a light source is necessary for them to learn to orient using a magnetic compass [90]. The early spatial behaviour of hatchling turtles is fundamentally influenced by innate patterns and imprinting; however, they subsequently develop the capacity to learn and remember the main direction or details of the entire route they have used, which they maintain throughout their lives. Adult turtles return repeatedly, once every few years, to the beaches or regions where they were born. This ability to navigate with such precision is characterised by a complex navigation strategy involving imprinting of local magnetic field characteristics based on the perception of intensity and inclination gradients (or a combination of these two parameters), probably combined with chemical and visual local cues at the end of the journey [91].

Terrestrial turtles also have excellent memory not only during migration [66] but also in other contexts, as demonstrated by discrimination and operant conditioning tasks, where they attend to visual cues [37,41,42]. This may also be important in spatial cue learning, where the marker directly indicates the target. An experiment on adult Aldabra tortoises (*Aldabrachelys gigantea*) and subadult Galapagos tortoises (*Chelonoidis* cf. *nigra*) was performed by Gutnick et al. [42]. The animals learned to distinguish colours in a two-choice discrimination task. Three of the available Aldabra tortoises were tested again after 9 years, and all of them immediately responded to the operant task but needed to relearn the discrimination task. In another study, red-footed tortoises (*Chelonoidis carbonarius*) were first trained to associate visual cues with particular foods. The animals were retested after 18 months and were still able to recall the cue colour associated with the reward [41]. Davis and Burghardt [37] studied the red-bellied cooters (*Pseudemys nelsoni*), semi-aquatic pond turtles that appear only in Florida. Nine turtles underwent a novel food acquisition task to examine their learning abilities and long-term memory. Animals were exposed to solve two-choice problem to choose the bottle with food as a reward. All experimental animals learned (achieving an average of 71% correct choices) during the training phase. Moreover, they have been able to remember the learnt task after at least two months, and their discrimination success after seven and a half months was still high (71–81% mean success rate). However, the animals differed in the number of trials to solve the task (training: 7–48 trials, after two months: 28–48 trials, after seven and a half months: 42–54 trials). The turtles remembered the discrimination task even after 36 months [38]. In all of these cases, it appears that turtles remember specific geographic locations or specific visual cues for months to years, although the mechanisms of initial acquisition may be different (innate preferences, imprinting, associative learning), and for much longer than the geckos remembered the relationships between visual cues in the Morris water maze.

A previous study demonstrated that leopard geckos exhibit highly individual navigation strategies in spatial tasks [31]. They show distinct preferences for different types of cues, typically relying on multiple proximal cues. However, when these proximal cues were manipulated—either removed or shifted—they were able to switch to distal room cues. Furthermore, they flexibly adjust their strategy in the absence of certain cues, which is indicative of allothetic orientation. Moreover, it is the species with temperature sex determination [67], and temperature during incubation has multiple effects on phenotype [92], including effects on brain development and hence cognition [93]. That influences the performance of the gecko, e.g., movement velocity, foraging activity, or antipredatory strategy [94,95]. Studies on lizards have demonstrated that those incubated at higher temperatures tend to have enhanced learning and memory capabilities. In the experiment with the Australian lizard (*Bassiana duperreyi*), it was found that individuals incubated at higher temperatures performed better in spatial learning tasks compared to those incubated at lower temperatures [96]. Incubation temperature can affect the neurodevelopment of reptiles, influencing brain morphology and neural connectivity, which in turn impacts cognitive functions [97]. Amiel and others [98] showed in their study that hatchlings of the scincid lizard (*Bassiana duperreyi*) incubated at lower temperatures had larger telencephalons and bigger neurons in their medial cortices. In contrast, hatchlings from higher temperatures had fewer but denser neurons, with more neurons in specific areas (the small-celled region of the medial cortex and the large-celled region of the medial cortex). These differences in forebrain development align with the observed variations in learning abilities between the two incubation groups of nesting lizards. The warm incubation conditions that produce enhanced cognitive performance also yield hatchlings that are larger and quicker than those from cooler incubation temperatures. Moreover, incubation at higher temperatures would reduce a hatchling’s vulnerability to predation [98]. In our study, we tested animals that were incubated at the optimal temperature of 28.5 °C, where more females were hatched and almost all animals developed. However, temperature fluctuates during incubation in our laboratory as well as in the natural conditions of the environment. It causes variability in behavioural features like activity and motivation that can both affect memory performance [98]. To investigate in detail the effect of temperature, it will be necessary to test animals in extreme temperatures. It is possible that even small changes in incubation temperature can initiate the individual variability in spatial learning that we see in our raw data [31]. However, this variability was filtered out statistically in all experiments, as the individual was always set as a random factor in linear mixed models. Our findings on memory in this species are quite strong, partly because a larger group was tested, not just a few individuals as in other papers [85,99], which again reduces the influence of individual variability on our findings.

## 5. Conclusions

Our study demonstrates that leopard geckos possess the ability to learn and retain spatial information, confirming the presence of long-term memory in this species. The geckos successfully remembered the location of the hidden platform after a two-month interval, showing an alternative cognitive strategy that should be advantageous in a less stable environment. Nevertheless, they are not able to retain the information for a longer time. If we compare the memory performance of geckos with other larger species of squamates, it is clear that large monitor lizards or beaded lizards can easily outperform small leopard geckos.

## Figures and Tables

**Table 1:** Results of the post hoc Tukey test for the LME model with the path length (distance) as the dependent variable, number of touches, the training phase as fixed effects, and ID of the animal as a random factor. All phases of the experiment were compared against all. We highlighted in bold those that are important for the demonstration of learning and memory. Abbreviations: S-Training (start of the training—first three training trials), F-Training (finish of the training—last three training trials), Memory test 1 (two months after the termination of initial training), S-Memory test 2 (first three retraining trials, four months after Memory test 1), F-Memory test 2 (last three retraining trials finishing the whole experiment).

Contrast	Estimate	S.E.	DF	t-Ratio	*p*-Value
S-Training—F-Memory test 2	0.962	0.111	538	8.7	<0.0001
**S-Training—F-Training**	0.4015	0.099	538	4.07	0.0005
S-Training—Memory test 1	0.275	0.101	538	2.71	0.0535
S-Training—S-Memory test 2	0.0136	0.098	538	0.14	0.9999
F-Memory test 2—F-Training	−0.5606	0.112	538	−5	<0.0001
F-Memory test 2—Memory test 1	−0.687	0.115	538	−5.98	<0.0001
**F-Memory test 2—S-Memory test 2**	−0.9484	0.111	538	−8.54	<0.0001
**F-Training—Memory test 1**	−0.1264	0.098	538	−1.28	0.7011
**F-Training—S-Memory test 2**	−0.3879	0.099	538	−3.91	0.001
**Memory test 1—S-Memory test 2**	−0.2614	0.102	538	−2.57	0.0769

**Table 2:** Coefficients from the LME model with the path length (distance) as the dependent variable, the number of touches, training phase as fixed effects, and animal ID as a random factor. Abbreviations: F-Training (Finish of the training—last three training trials), Memory test 1 (two months after the termination of initial training), S-Memory test 2 (first three retraining trials, four months after Memory test 1), F-Memory test 2 (last three retraining trials finishing the whole experiment).

	Value	95% CI (LL, UL)	S.E.	DF	t-Value	*p*-Value
Intercept	6.1418	5.970, 6.313	0.087	538	70.38	<0.0001
touches	0.0867	0.076, 0.097	0.005	538	15.85	<0.0001
F-Memory test 2	−0.962	−1.179, −0.745	0.111	538	−8.7	<0.0001
F-Training	−0.4015	−0.595, −0.208	0.099	538	−4.07	0.0001
Memory test 1	−0.275	−0.474, −0.076	0.101	538	−2.71	0.0069
S-Memory test 2	−0.0136	−0.207, 0.179	0.098	538	−0.14	0.8903

**Table 3:** Results of the post hoc Tukey test for the LME model with latency (stopwatch time) as the dependent variable, starting position, number of touches, the training phase, body weight as fixed effects, and animal ID as a random factor. All phases of the experiment were compared against all. We highlighted in bold those that are important for the demonstration of learning and memory. Abbreviations: S-Training (start of the training—first three training trials), F-Training (Finish of the training—last three training trials), Memory test 1 (two months after the termination of initial training), S-Memory test 2 (first three retraining trials, four months after Memory test 1), F-Memory test 2 (last three retraining trials finishing the whole experiment).

Contrast	Estimate	S.E.	DF	t-Ratio	*p*-Value
S-Training—F-Memory test 2	0.1198	0.13	536	0.92	0.8888
**S-Training—F-Training**	0.1885	0.11	536	1.71	0.4285
S-Training—Memory test 1	0.1166	0.11	536	1.05	0.8297
S-Training—S-Memory test 2	−0.3688	0.11	536	−3.31	0.0087
F-Memory test 2—F-Training	0.0687	0.12	536	0.59	0.977
F-Memory test 2—Memory test 1	−0.0033	0.12	536	−0.03	1
**F-Memory test 2—S-Memory test 2**	−0.4886	0.12	536	−4.22	0.0003
**F-Training—Memory test 1**	−0.072	0.1	536	−0.71	0.9537
**F-Training—S-Memory test 2**	−0.5573	0.1	536	−5.49	<0.0001
**Memory test 1—S-Memory test 2**	−0.4853	0.11	536	−4.63	<0.0001

## Data Availability

All data generated or analysed during this study are included in this published article in its Supplementary Materials (Files S1).

## Supplementary Materials

The following supporting information can be downloaded at: https://www.mdpi.com/article/10.3390/ani15142014/s1, File S1: Full dataset; File S2: Table S1: Results for latency; Table S2: Results for velocity; Table S3: Results of the post hoc test for velocity; Figure S1: The experimental arena for MWM testing adapted for small reptiles.
