# Supplementary material for: Memory in Leopard Geckos (Eublepharis macularius) in a Morris Water Maze Task
Source: Animals (Basel). 2025 Jul 8;15(14):2014. doi: 10.3390/ani15142014 (PMC12291964; doi:10.3390/ani15142014)
Supplement: Supplementary file 1 [file animals-15-02014-s001.zip › animals-3682275-supplementary/Online resources/File S2-Online Resource 2.pdf]

Online Resource 2

Memory in Leopard Geckos (Eublepharis macularius) in a Morris Water Maze Task

Eva Landová, Aleksandra Chomik, Barbora Vobrubová, Tereza Hruška Hášová, Monika Voňavková, Daniel Frynta and Petra Frýdlová *

Department of Zoology, Faculty of Science, Charles University, Viničná 7, 128 00 Prague, Czech Republic
*Correspondence: petra.frydlova@natur.cuni.cz; Tel.: +420-22195-1846

Table S1. Coefficients from the LME model with latency (stopwatch time) as the dependent variable, the start position, number of touches, training phase, and body weight as fixed effects and animal ID as a random factor. Abbreviations: F-Training (Finish of the training – last three training trials), Memory test 1 (two months after the termination of initial training), S-Memory test 2 (first three retraining trials, four months after Memory test 1), F-Memory test 2 (last three retraining trials finishing the whole experiment).

	Value	S. E.	DF	t-value	p-value
Intercept	1.1241	1.2011	536	0.9359	0.3497
Start position	-0.1071	0.0777	536	-1.3793	0.1684
Touches	0.0948	0.0056	536	16.8291	< 0.0001
F-Memory test 2	-0.1198	0.1301	536	-0.9211	0.3574
F-Training	-0.1885	0.1102	536	-1.7102	0.0878
Memory test 1	-0.1166	0.1106	536	-1.0542	0.2923
S-Memory test 2	0.3688	0.1113	536	3.3119	< 0.001
ln body weight	0.8262	0.3185	536	2.5941	0.0097

Table S2. Coefficients from the LME model with velocity as the dependent variable, the start position, number of touches, and training phase as fixed effects and animal ID as a random factor. Abbreviations: F-Training (Finish of the training – last three training trials), Memory test 1 (two months after the termination of initial training), S-Memory test 2 (first three retraining trials, four months after Memory test 1), F-Memory test 2 (last three retraining trials finishing the whole experiment).

	Value	S. E.	DF	t-value	p-value
(Intercept)	1.9568	0.0809	537	24.1841	< 0.0001
Start position	0.0669	0.0505	537	1.3239	0.1861
Touches	-0.0075	0.0037	537	-2.0347	0.0424
F-Memory test 2	-0.9859	0.0795	537	-12.4071	< 0.0001
F-Training	-0.3228	0.0673	537	-4.7967	< 0.0001
Memory test 1	-0.2453	0.0695	537	-3.5314	0.0004
S-Memory test 2	-0.5040	0.0671	537	-7.5084	< 0.0001

Table S3. Results of the posthoc Tukey test for the LME model with velocity as the dependent variable, start position, the number of touches, and training phase as fixed effects and animal ID as a random factor. All phases of the experiment

were compared against all. We highlighted in bold those which are important for the demonstration of learning and memory. Abbreviations: S-Training (Start of the training - first three training trials), F-Training (Finish of the training – last three training trials), Memory test 1 (two months after the termination of initial training), S-Memory test 2 (first three retraining trials, four months after Memory test 1), F-Memory test 2 (last three retraining trials finishing the whole experiment).

contrast	estimate	S.E.	df	t-ratio	p-value
S-Training – F-Memory test 2	0.9859	0.0795	537	12.4070	<0.0001
**S-Training – F-Training**	0.3228	0.0673	537	4.7970	<0.0001
S-Training – Memory test 1	0.2453	0.0695	537	3.5310	0.0041
S-Training – S-Memory test 2	0.5040	0.0671	537	7.5080	<0.0001
F-Memory test 2 – F-Training	-0.6631	0.0762	537	-8.7040	<0.0001
F-Memory test 2 – Memory test 1	-0.7406	0.0777	537	-9.5260	<0.0001
**F-Memory test 2 – S-Memory test 2**	-0.4819	0.0754	537	-6.3880	<0.0001
**F-Training – Memory test 1**	-0.0775	0.0654	537	-1.1840	0.7604
**F-Training – S-Memory test 2**	0.1811	0.0659	537	2.7470	0.0486
**Memory test 1 – S-Memory test 2**	0.2586	0.0676	537	3.8240	0.0014

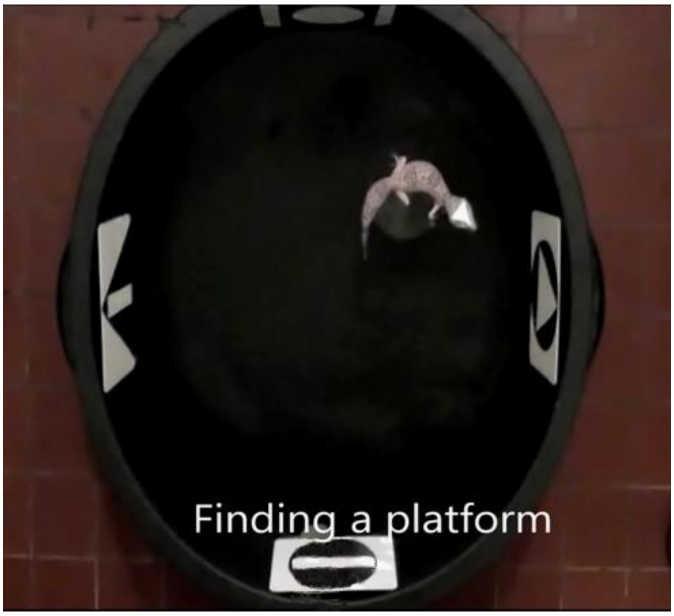

Figure S1. The experimental arena for MWM testing was adapted for small reptiles. The size of the arena was adjusted to the size of the experimental animals. A black plastic arena measured 51 cm in diameter, and the geckos had a total body length of 20 cm (snout-vent length of 12 cm). A transparent plastic cylinder was under the water as a platform. Four artificial markers (arena cues) were placed at the edge of the arena.
